# Cognitive, functional, physical, and nutritional status of the oldest old encountered in primary care: a systematic review

**DOI:** 10.1186/s12875-020-01128-7

**Published:** 2020-03-27

**Authors:** Emile Escourrou, Florence Durrieu, Bruno Chicoulaa, Julie Dupouy, Stéphane Oustric, Sandrine Andrieu, Virginie Gardette

**Affiliations:** 1 Département Universitaire de Médecine Générale, Faculté de Médecine Rangueil, Université Paul Sabatier Toulouse III, Toulouse, France; 2 UMR 1027 INSERM, Université Paul Sabatier Toulouse III, Toulouse, France; 3 Maison de Santé Pluri Professionnelle Universitaire La Providence, 1 avenue Louis Blériot, 31500 Toulouse, France; 4 Service d’épidémiologie, Centre Hospitalier Universitaire de Toulouse, Toulouse, France

**Keywords:** Aged 80 and over, Cognition, Nutritional status, Physical functional performance, Primary care, Systematic review

## Abstract

**Background:**

The oldest old (individuals over 90 years) are a fast-growing population. Characterizing their specificity would be helpful to adapt health care. This study aimed to characterize the cognitive, functional, nutritional, and physical status of individuals over 90.

**Methods:**

We conducted a systematic review of cross-sectional or cohort studies of individuals aged 90 years old or more, living at home or in a nursing home, in April 2018. Two reviewers selected eligible articles, extracted data, and evaluated the risk of bias (assessed by the Newcastle-Ottawa Scale).

**Results:**

The search strategy identified 3086 references; 35 articles were included referring to 8 cross-sectional and 27 longitudinal studies. Dementia was diagnosed in 30–42.9% of study participants, cognitive impairment in 12–50%, and 31–65% had no cognitive impairment. In terms of activities of daily living, 14–72.6% of individuals had no difficulty, 35.6–38% had difficulty, and 14.4–55.5% were dependent. For instrumental activities of daily living, 20–67.9% needed help. Regarding nutritional status, the Mini Nutritional Assessment Short Form mean score ranged from 10.3 (SD: 1.8) to 11.1 (SD: 2.4). Eight to 32% of individuals could not stand up from a chair, 19–47% could stand without the use of their arms; and 12.9–15% were not able to walk 4 m.

**Conclusions:**

These results suggest a heterogeneous population with a certain proportion of oldest old with a low level of disability. These findings suggest that a specific approach in the care of the oldest old could help prevent disability.

## Background

Population forecasts suggest that the population of 80 years old and over is likely to more than triple by the year 2050, from 126.5 million to 446.6 million [1].

This is a result of modifications in socio-environmental and biological factors during the human life course [2], with recent studies suggesting that some diets, economic status, the presence of a caregiver, are correlated with better aging [2–6]. Genetic signatures that predict the phenotypic outcome of exceptional long-living individuals are also identified [5, 6].

The oldest among the elderly are called the “oldest old”. Several definitions are proposed: the American Geriatric Society and the World Health Organization define the oldest old as individuals aged over 80 years, while the British Geriatrics Society uses 85 years as a threshold. In recent publications, the cut off has been fixed at 85 or 90 years and over [7–10].

The care of the oldest old is a growing topic in medical research and is a challenge for health care organizations. In this population, the aim of individual care is to allow a successful aging at home by preventing disability and loss of abilities [11, 12]. The desire to age at home, as well as population projections, create challenges for health care organizations, particularly in primary care.

Research on risk factors and preventive interventions for individuals 80 and over is limited. Despite an increasing number of cohorts studied in order to describe this population, such as the 90+ study, the Leiden 85-plus study, the Vitality 90+ study, the Newcastle 85+ study [e.g. 7–10], or recent literature on centenarians [13–15], few data describe the global status of individuals aged 90 and over.

We carried out a systematic review of the literature to better understand the characteristics of this population encountered in primary care in the coming years.

This study aimed to characterize the cognitive, functional, nutritional, and physical status of individuals over 90.

## Methods

This review was realized according to a systematic review process derived from the Preferred Reporting Items for Systematic reviews and Meta-Analyses (PRISMA) statement [16].

### Inclusion criteria

Cross-sectional or cohort studies of geriatric assessments, with individuals aged 90 years and over, were included. We set a cut off of individuals aged 90 and over in order to select a specific population of very old individuals. Participants had to live at home or in a nursing home. The studies had to assess at least one of the following outcomes (i.e. dimensions of a geriatric assessment): cognitive, functional, nutritional, or physical status, and had to be conducted with a minimum sample size of 100 participants. In order to ensure a sufficient precision of estimates, baseline data had to be described. In studies conducted on participants both under and over 90, the data for participants 90 and over had to be clearly identified and only those data were included. We restricted this review to high quality studies according to the Newcastle-Ottawa Scale (NOS), adapted to cross sectional reviews based on previous studies [17]. Data were only included for the studies allocated at least one star for 6 out of 7 items. There was no limitation on publication year.

### Exclusion criteria

We did not select studies that focused on a particular disease. Duplicate studies, or studies without any data about the studied outcomes and sample constitution, were not included. For studies that studied the same cohort, we included data only when different dimensions were assessed. Otherwise, we included only the most complete article.

### Search strategy and selection criteria

Preliminary to the extraction, two authors (E.E., F.D.) searched the websites World Health Organization, American Geriatric Society, European Geriatric Medicine Society, British Geriatric Society, French Geriatric and Gerontology Society, National Institute of Health, French National Authority of Health, and Google Scholar to delineate the subject and the search strategies.

Two authors (E.E., F.D.) applied the search strategies (See Additional file 1) to Medline, Cochrane Library, Pascal, and Web of Science related to mesh terms “aged 80 and over” “geriatric assessment” on April 24th, 2018. If available, the reference lists of previous similar literature reviews were carefully examined to manually identify potential eligible articles.

A two-step article screening was independently and blindly performed by the same two authors. The first selection was based on the title and/or abstract. Full texts were obtained for those studies that met the inclusion criteria, or when there was uncertainty. The second step was based on full text screening. Disagreements at each stage of selection were resolved by discussion, and through consultation with a third author (V.G.) if necessary.

### Data extraction

Data were extracted from the included studies by two authors (E.E. and F.D.), independently, using a pre-established standard assessment. The assessment included: author, year, country, cohort name, type of study (study design), settings, rate of participation, demographic characteristics, size of the sample, details of the gerontological assessment, and any or all of the following assessment results: cognitive, functional, nutritional, and physical status. For longitudinal studies, we used baseline data. Some studies did not provide adequate details on sex of the participants, in which case we provided aggregated results for men and women.

## Results

### Study selection

**Fig. 1:**
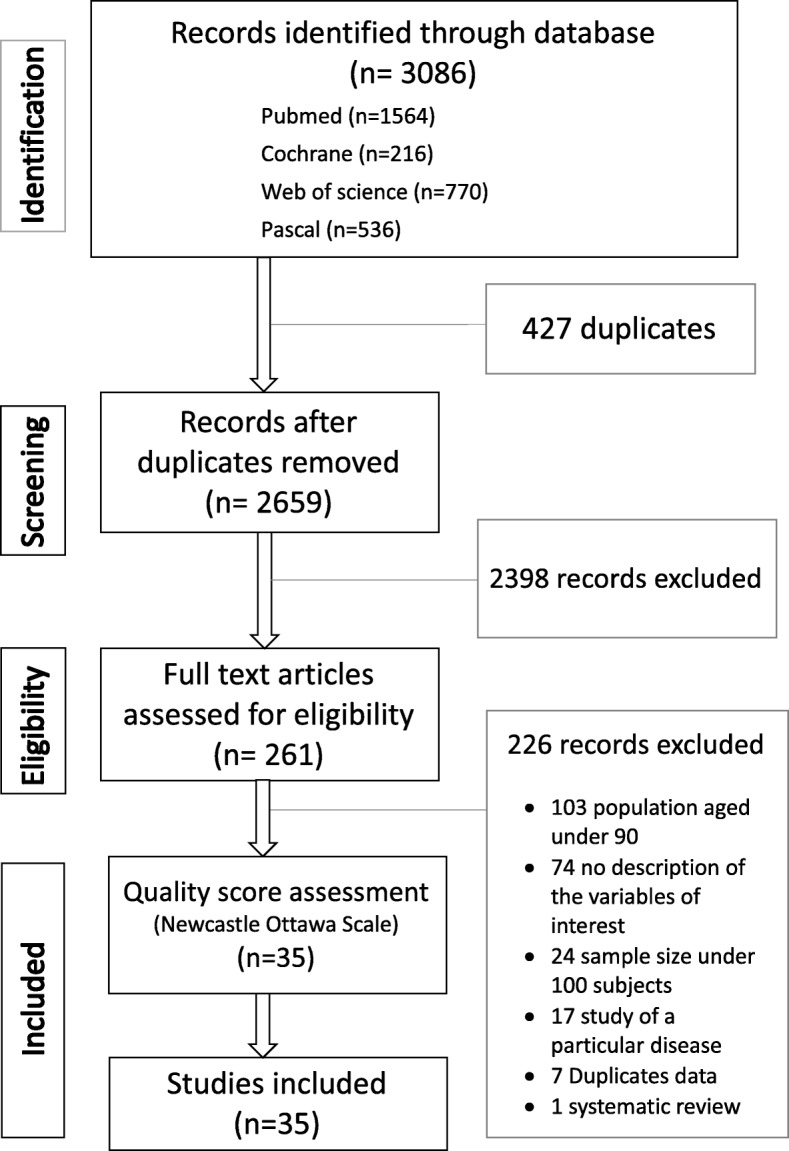
Flow chart of the review selection process

The search identified 3086 references. After the exclusion of duplicates, 2659 references were screened, and of these, 261 full text articles were reviewed (Fig. 1). Finally, 35 articles were included in this review and assessed for risk of bias [18–52]. No article was excluded after that assessment.

### Study characteristics (see Additional file 2)

Studies were conducted in 15 countries across four continents: Europe (*n* = 20) [20, 22, 23, 27–32, 37–40, 42–44, 47–49, 51], North America (*n* = 9) [18–20, 24–26, 41, 45, 46], Asia (*n* = 5) [33–36, 52], and Oceania (*n* = 1) [46].

Studies included individuals over 90 (*n* = 22) [18–25, 28, 30, 34, 35, 37, 38, 43–47, 49–51], individuals under and over 90 (only data from individuals over 90 were included) (*n* = 8) [29, 31, 32, 39, 40, 42, 48, 52], and centenarians (*n* = 5) [26, 30, 33, 36, 41].

The participation rate ranged between 47% [24] and 89% [18]; it was over 70% in more than 40% of studies. There were 27 longitudinal studies [18, 20–23, 25, 28, 29, 31, 34–49, 51, 52] and eight cross-sectional studies [19, 24, 26, 27, 30, 32, 33, 50] (three using databases from electronic medical files or administrative databases [29, 32, 50]).

Participants were living in a private home or nursing home (*n* = 27) [18–21, 23–29, 31, 33, 36–42, 44–49, 51], only living in private homes (*n* = 3) [34, 43, 50], and not specified (*n* = 5) [22, 30, 32, 35, 52].

Only four studies carried out an evaluation of all variables, cognitive, functional, physical and nutritional status [27, 32, 44, 48], providing a global approach of a same study population (Table 1).

**Table 1:** Results of the studies with an evaluation of the four characteristics of interest: cognitive, functional, physical and nutritional status

Author (year of study, country) [references]	Sample size [Living in: Home (H) and or Nursing Home (NH)]	Cognition	Functional status	Nutrition	Physical status
De Rango F et al. (2007, Italy) [27]	Age ≥ 90: 400 [H,NH]	**MMSE**^a^ (women; men) *Dementia* < 18: 74.6%; 47.2% *Moderate dementia* 18–23: 21.9%; 41.7% *Mild dementia or normal* > 23: 3.5%; 11.0%	**ADL** (women; men) Independence for the relevant activity Feeding 73.4%; 83.3% Transfer 46.1%; 64.1% Dress and undress 40.2%; 59.6% Use toilet 40.2%; 59.6% Bath or shower 26.6%; 35.9%	**BMI**^b^ Median (IR): 23.34 (5.1) Mean (SD): 23.68 (3.96)	**Hand grip**^c^ (kg) Women mean (SD): 10.85 (4. 65) Men mean (SD): 16.29 (8.84)
Herr M et al. (2010, France) [32]	Age ≥ 90: 512 [Not specified]	**MMSE**^a^ ≤ 26: 124 (26.3%) < 20: 124 (24.2%)	Need help for **ADL**: 167 (33%) Need help for **IADL**: 343 (67.9%)	**Weight loss and/or thinness** Women: 48 (14.2%) Men: 15 (9.3%)	**Lack of physical strength**: 307 (59.9%) **Low level of physical activit**y (IPAQ)^e^: 317 (61.9%)
Nybo H et al (1998, Denmark) [44]	Age ≥ 95: 2262 [H,NH]	**MMSE**^a^ *Mild dementia or normal* ≥ 23: 791 (34.9%) *Moderate dementia* 18–22: 575 (25.4%) *Severe dementia* 0–17: 398 (17.5%)	**ADL** Not disabled: 966 (42.7%) Moderately disabled: 807 (35.6%) Severely disabled: 458 (20.2%)	**BMI**^b^ < 22: 885 (39.1%) 22–27: 1069 (47.2%) ≥ 28: 222 (9%)	**Hand grip**^c^ Could complete 1649 (91.1%) Could not complete: 160 (8.8%) **Chair stand** Stand without use of arms: 909 Stand with use of arms: 680 Could not complete: 220 (9.5%)
von Heideken P et al (2000, Sweden) [48]	Age ≥ 90: 145 Age 90–94: 83 Age 95+: 62 [H,NH]	**MMSE**^a^ mean (range) *Age 90–94* Women: 23 (2–30) Men: 25 (16–29) *Age 95+* Women: 17 (0–28) Men: 22 (5–29) **Dementia** *Age 90–94* Women: 19 (31%) Men: 3 (14%) *Age 95+* Women: 25 (50%) Men: 3 (25%)	**P-ADL** Independent *Age 90–94* Women: 27 (44%) Men: 15 (71%) *Age 95+* Women: 10 (20%) Men: 5 (42%) **ADL** Independent *Age 90–94* Women: 7 (11%) Men: 6 (29%) *Age 95+* Women: 2 (4%) Men: 2 (17%)	**MNA**^d^ mean (range) *Age 90–94* Women: 22.5 (13.5–26.5) Men: 25 (19–27) *Age 95+* Women: 19 (10–27) Men: 25 (21–29)	**Usual gait speed** (m/s) Median (10th–90th perc) *Age 90–94* Women: 0.41 (0.18–0.69) Men: 0.51 (0.27–1.02) *Age 95+* Women: 0.41 (0.21–0.64) Men: 0.54 (0.19–0.81) **Fastest gait speed** (m/s) Median (10th–90th perc) *Age 90–94* Women: 0.75 (0.35–1.03) Men: 0.81 (0.39–1.33) *Age 95+* Women: 0.69 (0.46–1.06) Men: 0.92 (0.20–1.41) **Three chair stands** (sec) Median (10th–90th perc) *Age 90–94* Women: 11.9 (9–20.9) Men: 11.9 (8.3–26.3) *Age 95+* Women: 18.5 (10.3–24.7) Men: 12.2 (2.8–16)

*ADL* Activities of Daily Living, *BMI* Body Mass Index, *IADL* Instrumental Activities of Daily Living, *IPAQ* International Physical Activity Questionnaire, *MMSE* Mini Mental State Examination, *MNA* Mini Nutritional Assessment, *P-ADL* Performance of Activities of Daily Living.

^a^MMSE ranged from 0 to 30 (normal)

^b^BMI rates: Underweight ≤18.5, Normal weight = 18.5–24.9, Overweight = 25–29.9, Obesity = BMI of 30 or greater

^c^Hand Grip Strength: Individuals over 75 mean (SD) in kg: Women right hand: 19.0 (5), left hand: 17.0 (4) / Men right hand: 29.8 (9), left hand: 24.9 (7)

^d^MNA rates: Normal = 24–30, At risk of malnutrition = 17–23.5, Malnourished: < 17

^e^3 levels of activity were distinguished (low, moderate and high) according to time spent walking and doing moderate (for instance, carrying light loads, leisure bicycle ride, tennis) and vigorous activity (for instance, carrying heavy loads, digging, lifting a pack of 6 bottles or speed bicycle) during the past 7 days

### Dimensions of gerontological evaluation

#### Cognition

Cognitive status data (See Additional file 2) were provided in 25 studies [19, 23–28, 30–33, 35, 39–49, 51, 52], based on the Mini Mental State Examination (MMSE) (*n* = 22) [19, 23–28, 31–33, 35, 39, 40, 42–49, 51, 52], the Diagnostic and Statistical Manual of Mental Disorders, third or fourth edition, criteria for dementia (*n* = 6) [25, 40, 42, 47, 49, 51], and the Short Portable Mental Status Questionnaire (*n* = 1) [41]. One study did not provide this information [30].

Dementia was diagnosed in 30% [25] to 42.9% [42] of participants. Cognitive impairment was diagnosed in 12% [19] to 50% [41] of participants. No cognitive impairment was found in 31% [25] to 65.8% [45] of participants. The prevalence of dementia was more than 50% in studies where participants were over 95, and mean MMSE scores were also lower in these studies.

For articles reporting on populations with low MMSE, we searched the education level (if available) of the study population in order to suggest a link [24, 26, 27, 35]. (Table 2).

**Table 2:** Mini Mental State Evaluation results and education level for studies with low Mini Mental State Evaluation results in older people aged 90 and over

Author (year of study, country) [references]	Age (years: n) [Living in: Home (H) and or Nursing Home (NH)]	Education level	Mini Mental State Evaluation^a^ Mean (SD)
Cimarolli et al. (2014, USA) [24]	> 95: 119 [H,NH]	50% elementary school	16.48 (4.03)
Dai et al. (2008, USA) [26]	> 98: 244 [H,NH]	47% secondary school	16.2 (8)
De Rango et al. (2007, Italy) [27]	> 90: 400 [H,NH]	80% elementary school	Women: *< 18*: 74.6%; *18–23*: 21.9%; *> 23*: 3.5% Men: *< 18*: 47.2%; *18–23*: 41.7%; *> 23*: 11.0%
Ji-Rong et al. (2005, China) [35]	> 90: 682 [Not specified]	72% illiterate	15.54 (5.4)

^a^ Mini Mental State Evaluation ranged from 0 to 30 (normal)

#### Functional status

Functional status data were provided in 19 studies (See Additional file 2), and were based on Activities of Daily Living (ADL) (*n* = 19) [18, 22, 23, 26–28, 32, 36–39, 42–44, 46, 48, 49, 51, 52], and Instrumental ADL (IADL) (*n* = 7) [26, 28, 32, 36, 39, 46, 51].

The ADL Katz index was used, either the six-item version (*n* = 4) [18, 32, 36, 49, 52], the five-item version (*n* = 3) [22, 39, 44], or unspecified (*n* = 3) [23, 27, 43]. Four studies used the Barthel Index, either the 100-item version (*n* = 3) [28, 37, 38] or the 20-item version (*n* = 1) [42]. Two studies used another ADL index (ADL-staircase) [48], the Lawton 25 item B-ADL [46]. Three studies did not describe their scale [26, 51].

An eight-item IADL index (*n* = 3) [28, 32, 36], five-item IADL Katz Index (*n* = 1) [39], and the Bayer-IADL (*n* = 1) [46], were used, and one study did not provide this information [26, 51].

Based on these data, 14% [51] to 72.6% [49] of individuals were classed with the ADL scale as having no difficulty, 35.6% [44] to 38% [28] of individuals as “having difficulty”, and 14.4% [49] to 55.5% [18] as “dependent”. In addition, 20% [51] to 67.9% [32] needed help according to the IADL scale, and this was close to 90% [36] for centenarians.

As with cognition, studies with participants older than 95 [18, 23, 36, 39, 40, 42, 44, 46, 48] had lower ADL scores.

#### Nutritional status

Thirteen studies provided data on nutritional status (See Additional file 2) [20, 27–30, 32, 34, 37, 38, 41, 42, 44, 48], most commonly using Body Mass Index (BMI) (*n* = 9) [20, 27, 29, 30, 32, 37, 38, 41, 42, 44], followed by the Mini Nutritional Assessment Short Form (*n* = 2) [28, 34], the Mini Nutritional Assessment (*n* = 1) [48], the Malnutrition Universal Screening Tool (*n* = 1) [20], unintentional weight loss (*n* = 1) [32] and a serum albumin test (*n* = 1) [34].

The distribution of the study sample by BMI is as follows: *less than 18.5*: 1.9–12% [20, 30]; *between 18.5 and 24.9*: 58[30]-63.4% [29]; *between 25 and 29.9*: 15[30]-25% [30]; and *over 30*: 6 [30]-9.6% [29]. The mean BMI ranged from 23.68 (SD: 3.96) [27] to 25.1 (SD: 4.1) [42]. The Mini Nutritional Assessment Short Form mean score ranged from 10.3 (SD: 1.8) [34] to 11.1 (SD: 2.4) [28] (a score ≤ 11 indicates a risk of malnutrition).

#### Physical status

Nine studies provided data on physical status (See Additional file 2) [21, 23, 27, 32, 37, 43, 44, 48, 50], using mostly clinical tests, for example hand grip strength (*n* = 5) [21, 23, 27, 43, 44], ability to stand from a chair (*n* = 5) [21, 23, 37, 44, 48], and gait speed (*n* = 3) [23, 48]. The other tests were standing balance (*n* = 1) [21], a physical activity index based on the daily energy expenditure (kcal/kg/day) in the past 3 months (*n* = 1) [50] and the International Physical Activity Questionnaire (IPAQ) (*n* = 1) [32]. The results indicated that 32% [21] to 85% [23] of individuals over 90 could not stand up from a chair, and 19 [21] to 47% [23] could stand without the use of their arms. The overall mean grip strength (kilograms) was 14.5 (SD: 6.8) [43] to 16.1 (SD: 6.6) [23]; 10.85 for women and 16.29 for men [27]. Of the study population, 12.9% [21] to 15% [23] could not walk 4 m. A low level of physical activity was found in more than half of the study population.

## Discussion

### Commentary on results

Of 3086 references, we included 35 studies in our systematic review aiming to characterize the oldest old. The cognitive status was the most explored function (25/35), followed by functional status (19/35), nutritional status (13/35) and physical status (9/35).

The tests chosen for geriatric assessment were common tests used in most countries. However, there was a considerable variability in the tools used to assess each dimension with 3 tools for cognition (mostly represented by the Mini Mental State Evaluation), 6 for nutritional and physical status, and 9 for functional status. Such variability in the tests used in each of the 4 explored dimensions could bring variability in our results. We know for example that the prevalence of impairment may depend of the test used [53, 54]. The nutritional status was mainly described by BMI, whereas MNA-SF may be more accurate [55]. It could have been interesting to verify if sensitivity and specificity were identical when they were used for people aged 90 and over [56–58]. This gives rise to the need for a standardization in the assessments performed.

It has been demonstrated that some outcomes are associated with gender, in particular nutritional status (being female and unmarried determines poor nutritional status) [59] or physical status (difference in the mean hand grip strength, or mean gait speed) [60]. For those outcomes, we distinguished separate results for men and women if possible. Data were particularly different regarding physical outcomes, with lower performance in women.

Only four studies provided data on the four dimensions explored. Longitudinal and cohort studies appeared to focus mostly on cognitive and functional status. This limited the ability to provide several global evaluations of a sample of oldest old and to allow comparison between geographic areas for example.

For the studies with participants living at home and living in a nursing home, there was no information about the proportion of people living at home vs living in a nursing home. We cannot distinguish those 2 populations.

Cognitive impairment or functional disability was found half of the time. Nutritional status was abnormal for one quarter of the population. Physical status was abnormal for a third to half of the participants.

Our results indicated that individuals over 90 appeared as a heterogeneous population regarding cognitive, functional, physical, and nutritional status. Therefore, primary health care professionals may receive a range of patients, from those with preserved functions to those with dementia or a physical disability. Globally, the proportion of the oldest old with preserved functions is known. These findings are in line with forecasted trends for disabilities [61, 62].

### Comparison with younger elderly

#### Comparison with individual aged 65 and over

The prevalence of cognitive impairment (dementia excepted) appeared lower for individuals aged 65 and over [63, 64]. The nutritional status was comparable between populations of individuals aged 65 and over and individuals aged 90 and over [65]. Functional status was better among individuals aged 65 and over [66]. Hand grip strength decreased with age [67, 68], explaining a lower score in our result, in favor of higher prevalence of sarcopenia (See Additional file 3).

#### Comparison with frail individuals aged 65 and over

In a “younger” sample of 1108 frail individuals, cognitive function seemed higher [69]. Functional status was preserved while nutritional and physical status were altered but in a lower proportion compared to our results [69] (See Additional file 3).

The results of our review seem in a continuum with the data for individuals aged 65 and over [70].

### What are the implications for prevention and care for the oldest old?

Care plans for individuals need to take into account functions that can be preserved or maintained, as well as any disability already observed. A global evaluation would be helpful, such as the *Comprehensive Geriatric Assessment* recommended by the British Geriatrics Society for frail older people [71, 72], which includes a physical, psychological, and social assessment, from which a list of areas of need can be used to generate a care plan aimed at maintaining autonomy [73]. Person-centered care, after a global evaluation, and with efficient communication between all professionals, could improve healthcare quality and coordination, and thus, improve quality of life [71, 74–79].

As we have seen, there is more likely a continuum in the alteration of functions than a rupture with age. The solution may be to propose a care plan based on a global geriatric assessment earlier in the ageing trajectory to provide better maintenance of functions and autonomy among the oldest old in the future.

For the current population of oldest old, the implementation of such a care plan raises the question of its feasibility. The assessment of older patients is carried out in some geriatric day hospitals, especially for complex cases. New organizations for a geriatric assessment are proposed, to allow its realization in primary care [80, 81]. The geriatric assessment could be realized for example by a trained nurse in the patient’s home or in multi-professional primary care health centers with good results [82].

### Which interventions could be proposed?

Different interventions have been proposed to prevent cognitive dysfunction or disability [83–85]. These interventions are focused on cardiovascular disease management (e.g. nutrition, physical activity, and cognitive training) and oral supplementation (e.g. omega-3 for example) for individuals aged 60 or 70 and over. The benefits of such programs have to be studied for this specific population. The interventions showing a positive effect on preservation of function may have to be adapted to the physiopathological characteristics of the oldest old. A new approach should also be designed as suggested by Tischa et al. “*the establishment of collaborative networks between clinicians and designers, academia and industry is required to advance design for autonomous ageing*” [86].

### Strengths and limitations

Our study is the first to synthetize data relative to global descriptions of the oldest old. The studies included in this review are representative of the target population due to the decision to include older people living at home and in nursing homes, including those with loss of mobility. Data collection was generally performed in participants’ homes. This permitted the inclusion of individuals with mobility difficulties, and thus made samples more representative of the target population.

Our findings should be interpreted in light of several limitations. First, the studies included were from Western countries, with only four Asian studies and none from Africa, limiting the generalizability of findings. Second, when applicable, we used data from the base line and not from longitudinal surveys. This choice was made to prevent bias in longitudinal studies introduced by differential dropout [87]. Third, the NOS used to assess quality has been previously used in studies [88, 89] but not strictly methodologically validated. As far as we know, no other scale was available or recommended for cross sectional studies*.* Lastly, we decided to focus on 4 major dimensions, which are the most studied and have been used in intervention. It could have been interesting to complete with social, psychological, neurosensorial outcomes.

## Conclusion

These results suggest a heterogeneous population with a certain proportion of oldest old with preserved functions. It could encourage a specific approach in the care of the oldest old in order to prevent disability. These findings may inform an adaptation of health care services to address global and comprehensive care. This approach involves a better characterization of the population. Future research should evaluate interventions specific to this population.

## Supplementary information

**Additional file 1.** Different queries.

**Additional file 2.** Characteristics of included studies (ordered by continent (more represented by number of studies) and by dates in chronological order).

**Additional file 3.** Comparison between individuals aged 65 and over, frail individuals aged 65 and over, and individuals aged 90 and over regarding cognitive, functional, nutritional and physical status.

## Data Availability

Additional file 1 provides the search strategies applied to Medline, Cochrane Library, Pascal, and Web of Science; additional file 2 provides the characteristics of included studies; additional file 3 provides a comparison between individuals aged 65 and over, frail individuals aged 65 and over, and individuals aged 90 and over regarding cognitive, functional, nutritional and physical status. The datasets generated during this study (i.e. quality score assessment) are available on reasonable request.

## Abbreviations

MMSE: Mini Mental State Evaluation
ADL: Activities of Daily Living
IADL: Instrumental Activities of Daily Living
BMI: Body Mass Index
