# Supplementary material for: Cognitive, functional, physical, and nutritional status of the oldest old encountered in primary care: a systematic review
Source: BMC Fam Pract. 2020 Mar 27;21:58. doi: 10.1186/s12875-020-01128-7 (PMC7099824; doi:10.1186/s12875-020-01128-7)
Supplement: Supplementary file 1 — Additional file 1. Different queries. [file 12875_2020_1128_MOESM1_ESM.docx]

**Additional file 1:** Different queries

| **Pubmed** |
| --- |
| ((aged, 80 and over [MeSH Major Topic])) OR (nonagenarians [Title/Abstract]) OR (oldest old [Title/Abstract]) OR (aged 90 and over [Title/Abstract]))  AND  ((cognition [MeSH Terms]) OR (cognition disorders [MeSH Terms]) OR (Dementia/epidemiology*[MeSH Terms]) OR (Dementia/pathology*[MeSH Terms])  OR (physical function [Title/Abstract]) OR (sarcopenia [MeSH Terms])  OR (nutrition assessment [MeSH Terms]) OR (nutritional status [MeSH Terms])  OR (activities of daily living [MeSH Terms]) OR (Mobility Limitation*[MeSH Terms]) OR (health status [MeSH Terms])  OR (incidence [MeSH Terms])OR (prevalence [MeSH Terms])  OR (geriatric assessment [MeSH Terms])) |
| **Cochrane** |
| "oldest-old" in Title, Abstract, Keywords  or elderly in Title, Abstract, Keywords  or elder in Title, Abstract, Keywords  or "aged, 80 and over" in Title, Abstract, Keywords |
| **Pascal** |
| (abstract.\*:("aged, 80 and over") OR abstract.\*:(nonagenarians) OR abstract.\*:  ("oldest old")) AND (abstract.\*:(cognition) OR abstract.\*:(cognition disorders) OR  abstract.\*:(Dementia/epidemiology*) OR abstract.\*:(Dementia/pathology*) OR  abstract.\*:(physical function) OR abstract.\*:(sarcopenia) OR abstract.\*:(nutrition  assessment) OR abstract.\*:(nutritional status) OR abstract.\*:(activities of daily  living) OR abstract.\*:(Mobility Limitation*) OR abstract.\*:(health status) OR  abstract.\*:(incidence) OR abstract.\*:(prevalence) OR abstract.\*:(geriatric  assessment)) |
| **Web of science** |
| (TS=(oldest-old) OR TS=(nonangenarians) OR TS=(aged, 80 and over)) AND (TS=(cognition) OR TS=(physical function) OR TS=(health status) OR TS=(nutritional status) OR TS=(activities of daily living) OR TS=(geriatric assesment)) AND (TS=(longitudinal study*) OR TS=(cross sectional study*) OR TS=(cohort stydy*) OR TS=(epidemiologic study*)) |
