# Supplementary material for: Cognitive, functional, physical, and nutritional status of the oldest old encountered in primary care: a systematic review
Source: BMC Fam Pract. 2020 Mar 27;21:58. doi: 10.1186/s12875-020-01128-7 (PMC7099824; doi:10.1186/s12875-020-01128-7)
Supplement: Supplementary file 2 — Additional file 2. Characteristics of included studies (ordered by continent (more represented by number of studies) and by dates in chronological order). [file 12875_2020_1128_MOESM2_ESM.docx]

**Additional file 2:** Characteristics of included studies (ordered by continent (more represented by number of studies) and by dates in chronological order)

| **Author**  (year of study, country)  [references] | **Population**  **[Living in: Home (H) and or Nursing Home (NH)]** | **Sample size** | **Cognition** | **Functional status** | **Nutrition** | **Physical status** |
| --- | --- | --- | --- | --- | --- | --- |
| **NORTH AMERICA** | | | | | | |
| Boeve B *et al.*  (1999, USA)  [19] | All individuals residing in Rochester, MN, who were 90 to 99 years of age, inclusive, as of June 1, 1997.  [H,NH] | Age ≥ 90: 160  Data on 111 | **Issued from MMSE**  No cognitive impairment: 56 (50%)  Mild Cognitive Impairment: 13 (12%)  Dementia: 42 (38%) |  |  |  |
| Peltz CB *et al*  (2003, USA)  [45] | All surviving Leisure World Cohort Study participants aged 90 years and older were invited to join the 90+ Study.  [H,NH] | Age ≥ 90: 395 | **MMSE^A^**  Mean (Range): 26.3 (17-30)  *No cognitive impairment*: 260 (65.8%) (mean MMSE: 27.4 (27-30))  *Amnestic mild cognitive impairment*: 32 (8.1%) (mean MMSE: 26 (24-29))  *Nonamnestic mild cognitive impairment*: 32 (8.1%) (mean MMSE: 26.5 (24-30))  *Other cognitive impairment*: 71 (18%) (mean MMSE: 22.2 (17-30)) |  |  |  |
| Wister A *et al*  (2003, Canada)  [50] | Data in this analysis are from Statistics Canada’s Canadian Community Health Survey (CCHS). The total sample consists of approximately 135,500 respondents aged 12 and older living in private dwellings. Only respondents who were 90 to 99 years of age were analyzed, giving 626 nonagenarian respondents. living in private residences.  [H] | Age ≥90: 626 |  |  | **BMI**  Normal weight: 364 (61.3%)  Underweight: 48 (9%)  Overweight: 174 (29.3%)  Missing: 33 | **Physical and Activity**  Not limited: 81 (13.1%)  Limited: 535 (86.8%)  Missing: 10 |
| Berlau DJ *et al.* (2007, USA)  [18] | Individuals from The Leisure World Cohort Study still alive and aged 90 and older as of January 1, 2003 were eligible for participation in the 90+ Study.  [H,NH] | Age ≥ 90: 697  Age 90-94: 410  Age 95-99: 221  Age 100+: 66 |  | **ADL difficulty**  Entire group: 551 (79%)  *Age 90-94:* 290 (70.7%)  *Age 95-99:* 197 (89.1%)  *Age 100+:* 64 (97%)  **ADL dependency**:  Entire group: 383 (55.5%)  *Age 90–94:* 179 (43.7%)  *Age 95–99:* 147 (66.5%)  *Age 100+:* 61 (92.4%) |  |  |
| Dai T *et al.*  (2008, USA)  [26] | A population-based sample of centenarians and near-centenarians from phase III of the Georgia Centenarian Study (GCS, 2001–2008).  [H,NH] | Age ≥ 98: 244 | **MMSE^A^**  Mean (SD): 16.2 (8) | **IADL** mean (SD; Range)  26.0 (18.4; 0–58)^B^  **ADL** mean (SD; Range)  16.5 (8.1; 0–23)^B^ |  |  |
| Bullain S.*et al*. (2003-2009, USA)  [21] | Individuals from The Leisure World Cohort Study still alive and aged 90 and older as of January 1, 2003 were eligible for participation in the 90+ Study.  [H,NH] | Age ≥ 90: 629 |  |  |  | **4-m walk**  Normal: 148 (23.5%)  Subnormal time: 400 (63.6%)  Unable to perform: 81 (12.9%)  **5 chair stands**  Normal: 120 (19%)  Abnormal: 307 (48.8%)  Unable to perform: 202 (32%)  **Standing balance**  Full tandem ≥ 10s: 111 (17.6%)  Full tandem 1-9s: 162 (25.6%)  Semi tandem ≥ 10s: 81 (12.9%)  Side by side ≥ 10s: 93 (14.9%)  Unable to perform: 182 (28.9%)  **Grip** **Strength**  Normal: 134 (21.3%)  Abnormal: 371 (59%)  Unable to perform: 124 (19.7%) |
| Corrada M. M *et al.*  (2010, USA)  [25] | Individuals from The Leisure World Cohort Study still alive and aged 90 and older as of January 1, 2003 were eligible for participation in the 90+ Study.  [H,NH] | Age ≥ 90: 961 | **MMSE and DSM IV**  No dementia: 31%  Cognitively impaired not demented: 38%  Dementia: 30% |  |  |  |
| Martin P *et al.* (2012, USA)  [41] | The Exceptional Longevity Study recruited 152 centenarians in a Midwestern state. No participant was excluded but centenarians had to be able to answer assessment questions.  [H,NH] | Age ≥100:  180 | **SPMSQ**  Mean number of mistakes (SD): 3.97 (2.79)  50% mild levels of cognitive impairment  <4 errors: 48%  4-6 errors: 28%  7-10 errors: 24% |  | **BMI**  Underweight: 22%  Normal weight: 54%  Overweight: 20%  Obese: 4% |  |
| Cimarolli V R. *et al*.  (2014, USA)  [24] | 95 years old or older living in three diverse boroughs of New York City from a list provided by the Voters Registry and from three collaborating geriatric health care organizations.  [H,NH] | Age ≥ 95: 119 | **MMSE^A^**  Mean (SD): 16.48 (4.03) |  |  |  |
| **EUROPE** | | | | | | |
| Heeren T J *et al*.  (1989, Netherlands)  [31] | All available inhabitants of Leiden, aged 85 years or over on December 1, 1986 including subjects living in institutions.  [H,NH] | Age ≥ 90: 233 | **MMSE^A^**  < 24: 79 (33%) |  |  |  |
| Xie J *et al*  (1991, UK)  [51] | MRC CFAS is a population-based cohort study of individuals aged 65 years and over living in the community and in institutions.  [H,NH] | Age ≥90: 958 | **MMSE^A^**  *26–30:* 202(26%)  *22–25:* 207(27%)  *18–21:* 191(24%)  *0–17:* 182 (23%)  Missing data: 145 | **ADL** and **IADL^B^**  No disability: 121 (14%)  IADL disability only: 171 (20%)  IADL and ADL disability: 567 (66%)  missing data: 99 |  |  |
| von Strauss E *et al* (1993, Sweden)  [49] | Inclusion: all inhabitants aged of 90, living in the Kungsholmen district, Stockholm, Sweden.  [H,NH] | Age ≥90: 502 | **MMSE^A^**  Women <24: 63.8%  Men < 24: 47.1%  **DSM-III-R** dementia  197 (56.1%) | **ADL**  No disability: 72.6%  Partial disability: 13%  Disability: 14.4% |  |  |
| Nybo H *et al*  (1998, Denmark)  [44] | Danish 1905 cohort study  All Danes born in 1905 and living in Denmark.  [H,NH] | Age ≥ 95: 2262 | **MMSE^A^**  *≥23*: 791 (34.9%)  *18–22:* 575 (25.4%)  *0–17:* 398 (17.5%) | **ADL**  Not disabled: 966 (42.7%)  Moderately disabled: 807 (35.6%)  Severely disabled: 458 (20.2%) | **BMI^E^**  *<22:* 885 (39.1%)  *22–27:* 1069 (47.2%)  *≥ 28:* 222 (9%) | **Hand grip**  Could complete: 1649 (91.1%)  Could not complete: 160 (8.8%)  **Chair stand**  Stand without use of arms: 909  Stand with use of arms: 680  Could not complete: 220 (9.5%) |
| von Heideken P *et al* (2000, Sweden)  [48] | Half the 85-year-old population, and the total population aged 90 and ≥95 (range 95–103) in Umeå, Sweden who were measured in the Umeå 85+ Study.  [H,NH] | Age ≥ 90: 145  Age 85-90: 93 (excluded)  Age 90-94: 83  Age 95+: 62 | **MMSE^A^** mean (range)  *Age 90-94*  Women: 23 (2-30)  Men: 25 (16-29)  *Age 95+*  Women: 17 (0-28)  Men: 22 (5-29)  **Dementia**  *Age 90-94*  Women: 19 (31%)  Men: 3 (14%)  *Age 95+*  Women: 25 (50%)  Men: 3 (25%) | **P-ADL** Independent  *Age 90-94*  Women: 27 (44%)  Men: 15 (71%)  *Age 95+*  Women: 10 (20%)  Men: 5 (42%)  **ADL** Independent  *Age 90-94*  Women: 7 (11%)  Men: 6 (29%)  *Age 95+*  Women: 2 (4%)  Men: 2 (17%) | **MNA^I^** mean (range)  *Age 90-94*  Women: 22.5 (13.5-26.5)  Men: 25 (19-27)  *Age 95+*  Women: 19 (10-27)  Men: 25 (21-29) | **Usual gait speed**^J^ (m/s)  Median (10^th^-90^th^ perc)  *Age 90-94*  Women: 0.41 (0.18-0.69)  Men: 0.51 (0.27-1.02)  *Age 95+*  Women: 0.41 (0.21-0.64)  Men: 0.54 (0.19-0.81)  **Fastest gait speed**^J^ (m/s) Median (10^th^-90^th^ perc)  *Age 90-94*  Women: 0.75 (0.35-1.03)  Men: 0.81 (0.39-1.33)  *Age 95+*  Women: 0.69 (0.46-1.06)  Men: 0.92 (0.20-1.41)  **Three chair stands**^J^ (sec):  Median (10^th^-90^th^ perc)**:**  *Age 90-94*  Women: 11.9 (9-20.9)  Men: 11.9 (8.3-26.3)  *Age 95+*  Women: 18.5 (10.3-24.7)  Men: 12.2 (2.8-16) |
| Montesanto A *et al.* (2006, Italy)  [43] | Recruitment of subjects older than 90 years between 2002 and 2006, in the frame of two different European projects: the European Challenge for Healthy Aging (ECHA) (De Rango et al.2008) and the GEnetics of Healthy Aging (GEHA) (Skytthe et al.2011) projects.  [H] | Age ≥90:  505 | **MMSE^A^**  Mean (SD; range): 15.3 (6.2; 0-29) | **ADL^C^**  Mean (SD): 3.13 (1.98)  Range: 0-5 |  | **Hand grip strength** (kg)^D^  Mean (SD): 14.5 (6.8)  Range 1.0-42.0 |
| De Rango F *et al.* (2007, Italy)  [27] | All persons born before 1912 or earlier and with residence in all 409 Calabrian municipalities.  [H,NH] | Age ≥ 90: 400 | **MMSE^A^** Women  *Dementia <18:* 74.6%  *Moderate dementia 18–23:* 21.9%  *Mild dementia or normal >23:* 3.5%  **MMSE^A^** Men  *Dementia <18:* 47.2%  *Moderate dementia 18–23:* 41.7%  *Mild dementia or normal >23:* 11.0% | Independence for the relevant activity  **ADL** Women  Feeding 73.4%  Transfer 46.1%  Dress and undress 40.2%  Use toilet 40.2%  Bath or shower 26.6%  **ADL** Men  Feeding 83.3%  Transfer 64.1%  Dress and undress 59.6%  Use toilet 59.6%  Bath or shower 35.9% | **BMI^E^**  Median (IR): 23.34 (5.1)  Mean (SD): 23.68 (3.96) | **Hand grip** (kg)  Women mean (SD): 10.85(4.65)  Men mean (SD): 16.29 (8.84) |
| Molander L *et al* (2007, Sweden and Finland)  [42] | The Umeå 85+/GERDA study : half of all born in 1920, all born in 1915 and all born in 1910 or earlier living in 1 urban municipality in Västerbotten, Sweden, or 2 municipalities in Pohjanmaa, Finland, on January 1, 2005 and half of all born in 1922, all born in 1917 and all born in 1912 or earlier living in 5 rural municipalities in Västerbotten, Sweden, on January 1, 2007 were included.  [H,NH] | Age ≥ 90: 368  Age 85-89: 207 (excluded)  Age 90-94: 210  Age ≥ 95: 158 | **MMSE^A^** score mean (SD)  *Age 90:* 19.7 (7.8)  *Age ≥95:* 16.4 (8.1)  Dementia (**DSM IV**)  *Age 90:* 42.9%  *Age ≥95:* 56.3% | **ADL** score /20 mean (SD)^H^  *Age 90:* 16.1 (5.7)  *Age ≥95:* 13.1 (6.7) | **BMI^E^** mean (SD)  *Age 90:* 25.1 (4.1)  *Age ≥95:* 24.8 (4.3) |  |
| Cevenini *et al*  (2008, Italy)  [22] | Italian siblings of 90 years of age and older belonging to 552 sib-ships with at least two living members aged 90 years or older, recruited in Northern, Central and Southern Italy within the EU-funded project GEHA.  [Not specified] | Age ≥ 90: 1160 |  | **ADL**  Healthy/independent (MMSE ≥ 24 and ADL = 5) 331 (28.5%)  Unhealthy/dependent (MMSE < 24 or ADL < 5) 829 (71.5%) |  |  |
| Hajek A *et al*.  (2009, Germany)  [29] | Age ≥ 75 years, living at home, absence of dementia, and at least one contact with the GP during the last 12 months.  [H,NH] | Age ≥ 90: 104 |  |  | **BMI^E^**  *<18.5*: 2 (1.9%)  *18.5-24.9*: 66 (63.4%)  *25-30*: 26 (25%)  *> 30*: 10 (9.6%) |  |
| Herr M *et al*.  (2010, France)  [32] | Subjects were selected at random among the 2,100,000 recipients of a supplementary pension fund, AG2R La Mondiale (Paris, France).  [Not specified] | Age ≥ 90: 512 | **MMSE^A^**  *≤ 26:* 124 (26.3%)  *< 20:* 124 (24.2%) | Need help for **ADL**  167 (33%)  Need help for **IADL**  343 (67.9%) | **Weight loss and/or thinness**  Women: 48 (14.2%)  Men: 15 (9.3%) | **Lack of physical strength**^J^  307 (59.9%)  **Low level of physical activity**^M^ (IPAQ): 317 (61.9%) |
| Bonaccorsi G *et al*.  (2010, Italy)  [20] | 90 – 99 years old and living in the Mugello area  [H,NH] | Age ≥ 90: 321 |  |  | **BMI^E^**  *<18.5:* 12%  *18.5-21:* 25.2%  *22-26.9:* 40.5%  *28-29.9:* 13.1%  *>30:* 8.4%  **MUST**  low risk 74.8%  medium risk 11.5%  high risk 13.7% |  |
| Lucca U *et al*  (2010, Italy)  [39] | All registered individuals 80 years or older residing in the province of Varese, Italy were eligible for the study.  [H,NH] | Age ≥90: 719  Total: 1842  Age 80-84: 542  Age 85-89: 581 (excluded)  Age 90-94: 569  Age 95+: 150 | Mean (SD), median (25^th^-75^th^ percentiles)  **MMSE^A^**  *Age* 90-94: mean 20.6 (7.7)  Median 23 (16-26)  *Age 95+:* mean 16.8 (8.6)  Median 18 (11-24)  **Blessed-Information Memory Concentration test** (Individuals without MMSE)  *Age 90-94:* mean 10.4 (5.3)  Median 10 (5-13)  *Age 95+:* mean 10.0 (5.7)  Median 10 (6-14)  **Informant Questionnaire on Cognitive Decline in the Elderly**  *Age 90-94:* mean 4.0 (0.8)  median 4.0 (3.3-5)  *Age 95+:* mean 4.3 (0.7)  median 4.4 (3.5-5) | Mean (SD), median (25^th^-75^th^ percentiles)  **ADL^F^**  *Age 90-94:* mean 9.8 (9.6)  Median 6 (1-18)  *Age 95+:* mean 14.3 (9.7)  Median 15 (6-22)  **IADL** (%disability)  *Age 90-94:* mean 60.7 (35.1) Median 66 (27-97)  *Age 95+:* mean 77.9 (28.4)  Median 94 (63-100) |  |  |
| Christensen K *et al.*  (2010, Denmark)  [23] | All Danes born in 1915 who lived in Denmark at the time of the survey.  [H,NH] | Age 94-95: 1247 | **Cognitive composite score**  Mean (SD): 0.49 (3.6)  **MMSE^A^**  Mean (SD): 22.8 (5,6)  *28-30:* 277(23%)  *23-27:* 456(37%)  *18-22:* 281(23%)  *0-17:* 209 (17%) | **ADL^C^**  Mean (SD) 2.0 (0.8)  *≥ 3:* 265(17%)  *2-3:* 447(29%)  < *2*: 835(54%) |  | **Grip strength** (kg)  Mean (SD): 16.1 (6.6)  **Chair Stand**  Can without use of arms 573 (47%)  Can with use of arms 524 (43%)  Cannot: 27 (8%)  Missing data: 89 (5%)  **Gait Speed** (sec)  Mean (SD): 6.1 (3.1)  *>8:* 174 (21%)  *< 8*: 653 (79%)  Not able to walk: 181 (15%)  Missing data: 323 (18%) |
| Lisko I *et al.*  (2010, Finland)  [38] | The Vitality 90+ Study: all inhabitants aged ≥90 years of the city of Tampere, Finland  Participants in this study come from cohorts born in 1909–1910, 1912–1913, and 1920. Measurements for all cohorts were done at the ages of 90–91 years.  [H,NH] | Age ≥ 90: 569 |  | **ADL^G^**  *0–95*  Women: 240 (57.7%)  Men: 72 (47.1%)  *95-100*  Women: 176 (42.3%)  Men: 81 (52.9%) | **BMI^E^**  mean (SD)  Women: 25.5 (4.3)  Men: 25.0 (3.5)  **BMI^E^** Categories for women  *<20:* 30 (7.2%)  *20–24.9:* 185 (44.5%)  *25–29.9:* 141 (33.9%)  *≥30:* 60 (14.4%)  **BMI^E^** Categories for men  *<20:* 10 (6.5%)  *20–24.9:* 77 (50.3%)  *25–29.9:* 51 (33.3%)  *≥30:* 15 (9.8%) | **Chair stand**  Time sec median (IQR)  Women: 16 (13–24)  Men: 15 (11–20)  **Unable**  Women: 123 (30.4%)  Men: 29 (9.8%) |
| Formiga F *et al.* (2011, Spain)  [28] | Nonagenarian residents both in the community and institutionalized in Sant Feliu de Llobregat (Barcelona, Catalonia, Spain).  [H,NH] | Age ≥90: 186 | **MMSE^A^** score  Mean (SD): 21 (11)  <24: 102 (56%) | **ADL**^G^  Mean (SD): 60.8 (30)  < 61 (functional impairment): 71 (38%)  **IADL**^K^  Mean (SD): 2.1 (2.2) | **MNA-SF**^L^  Mean (SD): 11.1 (2.4)  MNA <11: 53 (28.4%) |  |
| Hazra N C et al. (2013, UK)  [30] | Aged 100 and older registered at general practices providing research quality data to Clinical Practice Research Datalink between January 1, 1990, and September 30, 2013.  [Not specified] | Age ≥ 100: 4236 | **Dementia**  Women: 1044 (12%)  Men: 125 (6%)  **Cognitive impairment or memory problems**  Women: 433 (5%)  Men: 82 (4%) |  | **BMI^E^** Women  Mean (SD): 23.1 (4.3)  *<18.5:* 407 (12%)  *18.5-24.9:* 2009 (58%)  *25-29.9:* 824 (15%)  *>30:* 216 (6%)  **BMI^E^** Men  Mean (SD): 23.8 (3.8)  *<18.5*: 46 (6%)  *18.5-24.9*: 474 (61%)  *25-29.9*: 211 (27%)  *>30:* 49 (6%) |  |
| Lucca U *et al.*  (2015, Italy)  [40] | The Monzino 80-plus  Study  All individuals 80 years or older residing in Castellanza, Gorla Maggiore, Gorla Minore, Marnate, Olgiate Olona, and Solbiate Olona and 85 years or older residing in Fagnano Olona on 2002 were eligible for the study and all individuals aged 90 years or older residing municipality of Gallarate on 2005 and to all those aged 100 years or older residing in the remaining municipalities of the province of Varese in 2009. In view of the low number of men aged 95 years or over, the study was further extended to include a random sample of men aged 95 to 99 years resident in the same municipalities as the centenarians, in the first 9 months of 2010.  [H,NH] | Age ≥90: 1250  Age 90–94: 705 (56.4%)  Age 95–99: 259 (20.7%)  Age 100+: 286 (22.8%) | **DSMIV** dementia:  Age 90-94 (n: 634): 42.5% (38.6–46.4)  Age 95-99 (n: 255): 55.3% (53.2–57.5)  Age 100+ (n:267): 65.9% (59.9–71.6)  **MMSE**  Mild Dementia:  *90-94:* 9.6% (7.4–12.2)  *95-99*: 8% (6.9–9.1)  *100+:* 6% (3.5–9.6)  Moderate dementia:  *90-94:* 21.2% (18.1–24.6)  *95-99:* 28.4% (26.6–30.2)  *100+:* 26.6% (21.4–32.3)  Severe dementia:  *90-94:* 11.6% (9.3–14.4)  *95-99:* 19% (17.2–20.7)  *100+*: 33.3% (27.7–39.3) |  |  |  |
| Lisko I *et al.*  (2017, Finland)  [37] | The Vitality 90+ Study: all inhabitants aged ≥90 years of the city of Tampere, Finland.  [H,NH] | Age ≥ 90: 425 |  | **ADL disability** Women  No incident disability: 108 (37.1%)  Incident disability: 44 (15.1%)  Deceased: 138 (47.4%)  **ADL disability** Men  No incident disability: 38 (28.4%)  Incident disability: 10 (7.5%)  Deceased: 86 (64.2%) | **BMI^E^** women  *<20:* 19 (6.8%)  *20–24.9:* 125 (45.0%)  *25–29.9:* 101 (36.3%)  *≥30:* 33 (11.9%)  **BMI^E^** men  *<20*: 7 (5.8%)  *20–24.9*: 62 (51.7%)  *25–29.9:* 39 (32.5%)  *≥30*: 12 (10.0%) |  |
| Skoog, J et al.  (NA, years of publication 2017, Sweden)  [47] | All 97-year-olds living in Gothenburg, Sweden, born between July 1, 1901, and December 31, 1909.  [H,NH] | Age ≥ 97:  591:  484 women, 107 men | **MMSE^A^** mean  age 97: 17.3  age 99:16.2  age 100: 17.0 |  |  |  |
| **ASIA** | | | | | | |
| Inagaki H *et al*. (2003, Japan)  [33] | Centenarians living in 23 wards of Tokyo from lists of centenarians, published by the Japanese Ministry of Health, Labor and Welfare in 2000 and 2001.  [H,NH] | Age ≥ 100: 232 | **MMSE^A^**  Mean (SD): 22.3 (3.32) |  |  |  |
| Ji-Rong Y *et al*.  (2005, China)  [35] | Participants, older than 90 years, living in the 21 towns of Dujiangyan, Sichuan Province, China.  [Not specified] | Age ≥ 90: 682 | **MMSE^A^**  Mean (SD): 15.54 (5.4)  0-18: 429 (62.9%) |  |  |  |
| Ji L *et al.*  (2005, China)  [34] | Participants, older than 90 years, living in the 21 towns of Dujiangyan, Sichuan Province, China.  [H] | Age ≥ 90: 632 |  |  | **Serum albumin**^N^  Mean g/l (SD): 42.9 (3.4)  **MNA-SF** ^l^  Mean (SD): 10.3 (1.8)  Normal nutritional status: 151 (23.9%)  At risk for malnutrition (MNA 8-11): 445 (70.4%)  Malnourished (MNA < 8): 36 (5.7%) |  |
| Kim H *et al.*  (2006, south Korea)  [36] | Korean National Statistical Office (KNSO) Centenarian Survey  The survey identified the current status of the centenarian citizens within each city and province, regional officials conducted a field survey to interview the centenarians and their caregivers.  [H,NH] | Age ≥100: 796 |  | **ADL**  Non-disabled: 324 (41.2%)  Disabled: 463 (58.8%)  **IADL**  Non-disabled: 72 (9.1%)  Disabled: 715 (90.9%) |  |  |
| Zeng Y, *et al.*  (2008, China)  [52] | Participants aged 80–105 years from the 1998 and 2008 waves of the Chinese Longitudinal Healthy Longevity Surveys (CLHLS): nationwide survey done in a randomly selected half of the counties and cities in 22 of the 31 provinces, covering about 85% of the total population of China.  [Not specified] | Age ≥ 90: 2 cohorts (A and B):  **A**: date of birth 1899–1908: n=2896  **B**: date of birth: 1909–18 n=4338 | **MMSE^A^** mean (SD)  A: 20.62 (7.93)  B: 17.41 (9.62) | **ADL disability** mean (SD) **^B^**  A: 0.94 (1.62)  B: 0.74 (1.55) |  |  |
| **AUSTRALIA** | | | | | | |
| Sachdev P *et al* (2006, Autralia)  [46] | Cohort of individuals aged 95 years or above who are examined biannually and recruited into the brain donor program. Includes individuals aged ≥ 95 years who agree to participate, except those suffering from a terminal illness.  [H,NH] | Age ≥ 95: 200 | **MMSE^A^** n=186  Mean (SD): 21.1 (6.1)  54% with dementia | **ADL**^H^  Mean (SD): 13.4 (6.8)  **IADL**^K^  Mean (SD): 4.46 (3.3) |  |  |

ADL = Activities of Daily Living ; BMI = Body Mass Index ; IADL = Instrumental Activities of Daily Living ; IPAQ = International Physical Activity Questionnaire ; MMSE = Mini Mental State Examination ; MNA = Mini Nutritional Assessment ; MNA – SF = Mini Nutritional Assessment Short Form ; P-ADL = Performance of Activities of Daily Living ; SPMSQ = Short Portable Mental Status Questionnaire.

^A^ MMSE ranged from 0 to 30 (normal), the cut off for the status of cognitive function differed between studies (mid cognitive impairment, dementia)

^B^ These studies did not describe their scale

**^C^** ADL score > 5: autonomous

^D^ Hand Grip Strength: Individuals over 75 mean (SD) in kg: Women right hand: 19.0 (5), left hand: 17.0 (4) / Men right hand: 29.8 (9), left hand: 24.9 (7)

^E^ BMI rates: Underweight ≤ 18.5, Normal weight = 18.5–24.9, Overweight = 25–29.9, Obesity = BMI of 30 or greater

^F^ Scores ranged between 0 and 30, the lowest indicating no degree of dependence

^G^ ADL ranged between 0 (totally dependent) to 100 (fully independent)

^H^ ADL ranged between 0 (totally dependent) to 20 (fully independent)

^I^ MNA rates: Normal = 24-30, At risk of malnutrition = 17-23.5, Malnourished: < 17

^J^ No cut off for these tests

^K^ IADL ranged between 0 (totally dependent) to 8 (fully independent)

^L^ MNASF rates: Normal = 12-14, At risk of malnutrition = 8-11, Malnourished: < 7

^M^ 3 levels of activity were distinguished (low, moderate and high) according to time spent walking and doing moderate (for instance, carrying light loads, leisure bicycle ride, tennis) and vigorous activity (for instance, carrying heavy loads, digging, lifting a pack of 6 bottles or speed bicycle) during the past 7 days.

^N^  Albumin normal range: 35-55 g/liter
