# Supplementary material for: Cognitive, functional, physical, and nutritional status of the oldest old encountered in primary care: a systematic review
Source: BMC Fam Pract. 2020 Mar 27;21:58. doi: 10.1186/s12875-020-01128-7 (PMC7099824; doi:10.1186/s12875-020-01128-7)
Supplement: Supplementary file 3 — Additional file 3. Comparison between individuals aged 65 and over, frail individuals aged 65 and over, and individuals aged 90 and over regarding cognitive, functional, nutritional and physical status. [file 12875_2020_1128_MOESM3_ESM.docx]

**Additional file 3**: Comparison between individuals aged 65 and over, frail individuals aged 65 and over, and individuals aged 90 and over regarding cognitive, functional, nutritional and physical status.

| **Status** | **Individuals 65+** | **Individuals 90+** | **Frail individuals 65+** |
| --- | --- | --- | --- |
| **Cognitive** | Cognitive impairment 14.2-18% [63,64] | MMSE ^a^ mean (SD)  *Lower: 15.3 (SD: 6.2)* [43]  *Higher: 26.3 (range 17-30)* [45]  Dementia 30-42.9% [25,42]  Cognitive impairment 12-50% [19,41]  No cognitive impairment 31-65% [25,45] | MMSE ^a^ mean: 24.6 (SD: 4.9) [69] |
| **Functional** | Dependent for at least one item of ADL: 34.6% [65]  Dependent for at least one item IADL: 53.5% [65] | Dependent for at least one item of ADL: 35.6-38% [44,28]  Dependent for at least one item of IADL: 20-67.9% [51,32]  ADL **^b^** mean (SD)  *Lower: 2.0 (0.8)* [23]  *Higher: 3.13 (1.98)* [43]  IADL ^c^ mean (SD) 2.1 (2.2) [28] | ADL **^b^** mean (SD): 5.5 (1.0) [69]  IADL ^c^ mean (SD): 5.6 (2.4) [69] |
| **Nutritional** | Risk of malnutrition: 67% [66]  Malnutrition: 5% [66] | MNA-SF ^d^ mean 10.3 (SD: 1.8) to 11.1 (SD: 2.4): risk of malnutrition [34,28] | Risk of malnutrition: 39.5% [69]  Malnutrition: 8% [69] |
| **Physical** | Hand grip strength ^e^ mean (SD) kg:  *70-79 years: 25.5 (7.96)* [67]  *80 and over: 21.1 (6.78)* [68] | Hand grip strength ^e^ mean (SD) kg:  *Lower: 14.5 (6.8)* [43]  *Higher: 16.1 (6.6)* [23] | Hand grip strength ^e^ mean (SD) kg:  20.6 (8.2) [69] |

ADL = Activities of Daily Living; IADL = Instrumental Activities of Daily Living; MMSE = Mini Mental State Examination; MNA – SF = Mini Nutritional Assessment Short Form.

In square brackets [XX]: references.

^a^ MMSE ranged from 0 to 30 (normal), the cut off for the status of cognitive function differed between studies (mid cognitive impairment, dementia)

**^b^** ADL ranged between 0 (totally dependent) to 6 (fully independent)

^c^ IADL ranged between 0 (totally dependent) to 8 (fully independent)

^d^ MNASF rates: Normal = 12-14, At risk of malnutrition = 8-11, Malnourished: < 7

^e^ Hand Grip Strength: Individuals over 75 mean (SD) in kg: Women right hand: 19.0 (5), left hand: 17.0 (4) / Men right hand: 29.8 (9), left hand: 24.9 (7)
